# Design and implementation of a metagenomic analytical pipeline for respiratory pathogen detection

**DOI:** 10.1186/s13104-024-06964-9

**Published:** 2024-10-03

**Authors:** Pablo Alessandro B Viana, Diogo Antonio Tschoeke, Laise de Moraes, Luciane Amorim Santos, Manoel Barral-Netto, Ricardo Khouri, Pablo Ivan P Ramos, Pedro Milet Meirelles

**Affiliations:** 1 Institute of Biology, Federal University of Bahia (UFBA), Salvador, Brazil; 2 Center for Data and Knowledge Integration for Health (CIDACS), Gonçalo Moniz Institute, Oswaldo Cruz Foundation (Fiocruz), Salvador, Bahia Brazil; 3 Health Systems Engineering Laboratory, Alberto Luiz Coimbra Institute of Graduate Studies and Engineering Research (COPPE), Federal University of Rio de Janeiro (UFRJ), Rio de Janeiro, Brazil; 4 Institute of Biology, Universidade Federal do Rio de Janeiro (UFRJ), Rio de Janeiro, RJ Brazil; 5 Laboratory of Precision Medicine and Public Health (MESP 2), Gonçalo Moniz Institute, Oswaldo Cruz Foundation (Fiocruz), Salvador, Bahia Brazil; 6 Federal University of Bahia School of Medicine, Salvador, Bahia, 41745-715 Brazil; 7 National Institute for Interdisciplinary Transdisciplinary Studies in Ecology and Evolution (IN-TREE), Salvador, Brazil

**Keywords:** Respiratory pathogens, Metagenomics, Bioinformatics pipeline

## Abstract

**Objective:**

We developed an in-house bioinformatics pipeline to improve the detection of respiratory pathogens in metagenomic sequencing data. This pipeline addresses the need for short-time analysis, high accuracy, scalability, and reproducibility in a high-performance computing environment.

**Results:**

We evaluated our pipeline using ninety synthetic metagenomes designed to simulate nasopharyngeal swab samples. The pipeline successfully identified 177 out of 204 respiratory pathogens present in the compositions, with an average processing time of approximately 4 min per sample (processing 1 million paired-end reads of 150 base pairs). For the estimation of all the 470 taxa included in the compositions, the pipeline demonstrated high accuracy, identifying 420 and achieving a correlation of 0.9 between their actual and predicted relative abundances. Among the identified taxa, 27 were significantly underestimated or overestimated, including only three clinically relevant pathogens. We also validated the pipeline by applying it to a clinical dataset from a study on metagenomic pathogen characterization in patients with acute respiratory infections and successfully identified all pathogens responsible for the diagnosed infections. These findings underscore the pipeline’s effectiveness in pathogen detection and highlight its potential utility in respiratory pathogen surveillance.

**Supplementary Information:**

The online version contains supplementary material available at 10.1186/s13104-024-06964-9.

## Introduction

The rapid expansion of genomic and metagenomic data has significantly increased the complexity of analyzing and interpreting large-scale nucleotide sequences. Researchers face substantial challenges in managing and integrating diverse datasets with the development of various sequence formats and software tools [1]. This issue is further compounded by the frequent need to process complex sequence files using disparate tools that often lack interoperability.

There has been a growing trend toward developing custom bioinformatics pipelines to address these challenges to streamline data processing and enhance analytical efficiency. Methods traditionally used in scientific research to manage and analyze genomic data commonly employ ad-hoc scripting, which often lead to redundant code and inefficiencies, complicate data organization and increase the potential for errors in data processing and interpretation [2]. The increasing incorporation of metagenomics into clinical and surveillance practices has called for more standardized and simpler computational tools and routines, also demanding reproducible results across laboratories.

Our work focused on developing an in-house bioinformatics pipeline for pathogen detection in metagenomic data. It was built on established and open-source tools to provide a robust, scalable, customizable, and efficient solution for identifying respiratory pathogens. We employed this method in the context of the ÆSOP initiative, which combines digital and molecular approaches to improve outbreak prediction and surveillance in Brazil [3]. By leveraging high-performance computing resources and integrating advanced analytical methods, our pipeline addresses the need for accurate and rapid pathogen detection, enhancing the capability to monitor and respond to infectious disease threats.

## Materials and methods

### Pipeline description

Our bioinformatics pipeline integrates widely recognized open-source tools for each step. Fastp [4] was employed for adapter trimming, quality filtering, and preprocessing to enhance data quality. HISAT2 [5] and Bowtie2 [6] were employed for human read removal, combining Bowtie2’s broad alignment capabilities with HISAT2’s precision in handling spliced regions, ensuring thorough decontamination. This step is particularly crucial in clinical samples, where human DNA is abundant. Effective decontamination reduces the risk of false positives in microbial analysis, leading to more accurate identification and quantification of microbial taxa. For taxonomic classification, we used Kraken2 [7], which applies exact k-mer matching and the LCA algorithm for fast, accurate classification of large metagenomic datasets. We also used Bracken [8] statistically refining Kraken2’s output, re-estimating species-level abundance, to improve accuracy of the microbial composition.

### Pipeline steps

**Fig. 1:**
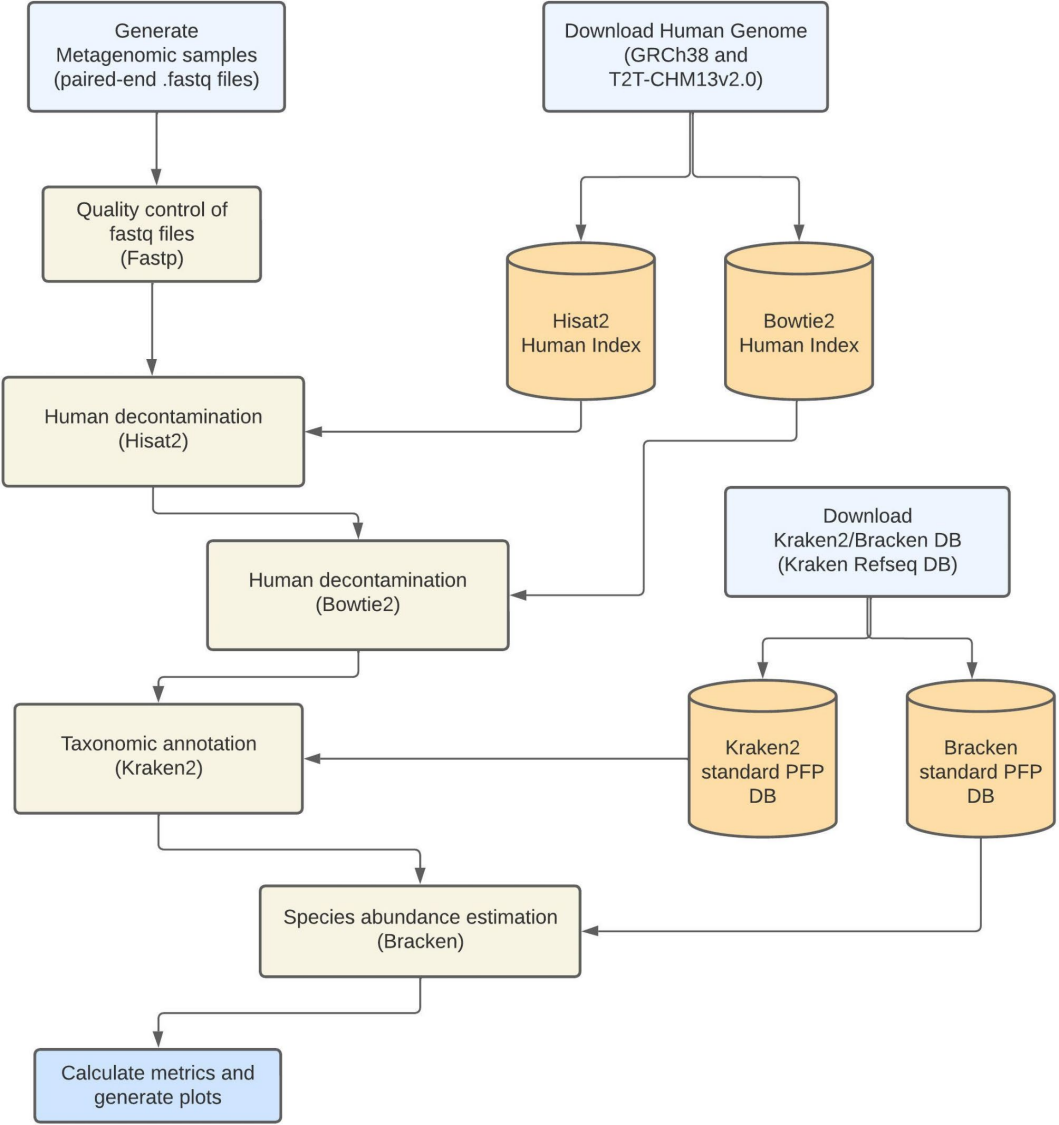
Overview of the proposed bioinformatics pipeline for pathogen detection in metagenomes from human nasopharyngeal swab samples

The proposed pipeline follows the workflow shown in Fig. 1. Details of each step are provided below:

1. **Obtaining and indexing reference databases**: Before executing our pipeline, we created HISAT2 and Bowtie2 indexes using GRCh38 and T2T-CHM13v2.0 as human reference genomes. For taxonomic classification, we used the standard plus PFP database provided by Kraken2, including genomes from NCBI RefSeq, covering archaea, bacteria, viruses, plasmids, humans, protozoa, fungi, and plants (see data availability).
2. **Sequence Quality Control**: To ensure the integrity and quality of the sequencing data, we conducted a rigorous quality control process using the Fastp tool (version 0.20.1). This process included trimming adapter sequences, filtering out reads shorter than 50 bp, discarding reads with a Phred quality score below 20, and removing reads containing more than two ambiguous (N) bases.
3. **Contaminant Cleanup**: A critical component of our methodology was the identification and removal of potential contaminant sequences. To achieve this, metagenomic sequences were mapped against a customized human index using a combination of HISAT2 (version 2.2.1) and Bowtie2 (version 2.4.4), with mapping QC done using Samtools (version 1.18) [9]. HISAT2 was run with default parameters, and the resulting alignments were saved in a SAM file. Sequences aligning to the human references were flagged and removed from the dataset using Samtools, specifically employing the *-f 13* parameters to retain read pairs where neither mate aligned to the reference. Subsequently, Bowtie2 was utilized with the *--very-sensitive-local* parameter to ensure a more sensitive alignment, also outputting the results to a SAM file. The unaligned pairs were again recovered using Samtools with the same parameters, ensuring the comprehensive removal of human sequences while preserving non-host reads.
4. **Taxonomic Annotation**: Taxonomic annotation was performed using Kraken2 (version 2.1.2), a high-throughput tool that classifies sequencing reads based on k-mer matches against an extensive reference database. Following Kraken2’s initial classification, species-level abundance estimates were refined using Bracken (version 2.9). Bracken leverages the classification data generated by Kraken2, applying a probabilistic model to reassign reads more accurately to taxonomic categories, thereby providing a higher resolution and more precise quantification of species abundance within the samples.

### Mock metagenomes

To implement and evaluate the pipeline, we defined nine metagenome compositions based on actual samples and predefined throat taxonomic profiles from the MeSS tool (https://github.com/metagenlab/MeSS), including information from all taxonomic levels using TaxonKit [10]. For each composition, taxa abundances were randomly generated using a log-normal distribution, with human DNA content ranging from 70 to 90%, reflecting a realistic scenario for analyzing respiratory infections (Table S1).

Metagenomes were generated using InSilicoSeq [11], producing ten simulated samples (i.e., mock metagenomes) for each composition. By varying the generator’s random seed, each sample within the same composition serves as a replica, with different reads randomized from the same genomes. All 90 mock metagenomes were created with error profiles characteristic of Illumina MiSeq sequencing, with each sample consisting of 1 million paired-end reads of 150 bp (Table S2).

### Critical pathogen definition

A pathogen taxa list (Table S3) was defined using a comprehensive list derived from the CZID, the Illumina RPIP pathogen list, the Illumina VSOP list, CDC outbreak reports, and WHO priority pathogen list reports (see data availability). Then, the CDC and WHO reports were manually curated to extract information on critical and high-priority pathogen species and families, which were categorized under the priority columns in Table S3. These priority pathogens represent what we refer to as critical pathogens. This list of critical pathogens was used to filter the taxa predicted by the pipeline, ensuring that our results focus on the most relevant viruses and bacteria.

### Empirical validation on clinical dataset

To further assess the accuracy of our pipeline, we applied it to a clinical dataset from a study on metagenomic pathogen characterization in patients with acute respiratory infections [12]. The dataset, consisting of 55 samples deposited in the NCBI SRA database (BioProject accession PRJNA540900), encompassed approximately 500GB of data. We processed all samples through the full pipeline and compared the identified organisms and the final clinical diagnoses, which indicated the pathogens responsible for each patient’s infection.

### Statistical analysis

This study’s statistical analyses and data visualizations were conducted using R (version 4.3.0). The following R packages were employed: *tidyverse* (version 2.0.0) for data manipulation and visualization, *scales* (version 1.2.1) for color management and scaling, *ggpubr* (version 0.6.0) for publication-ready figures, *ggrepel* (version 0.9.3) for improved label placement in plots, *patchwork* (version 1.1.2) for combining multiple plots. To assess the accuracy of our pipeline in predicting taxonomic profiles, we performed a Pearson correlation analysis by comparing the observed relative abundance of each taxon in the original samples with the predicted relative abundance generated by our pipeline.

## Results

To study the performance of the pipeline to identify pathogenic taxa in metagenomic samples, we produced 90 mock metagenomes simulating human nasopharyngeal swab samples and comprising 56 GB of metagenomic sequences. The pipeline efficiently processed these large datasets, thoroughly decontaminating the samples for microbial analysis by consistently removing or classifying more than 99.8% of human reads across all compositions and replicas. On average, the pipeline successfully taxonomically identified approximately 94% of the clean metagenomic reads in all samples (Table S2).

### Execution time

All analyses were executed in a HPC environment, utilizing 32 vCPUs and 512 GB of RAM. To maximize efficiency, we ran four parallel analysis threads. The average processing times per sample were as follows: (i) 0.4 min for sequence quality control and cleaning, (ii) 1.5 min for removing contaminating human sequences, and (iii) 2 min for taxonomic annotation. The complete analysis of each sample took approximately 4 min from raw data to results.

### Pathogen detection

**Fig. 2:**
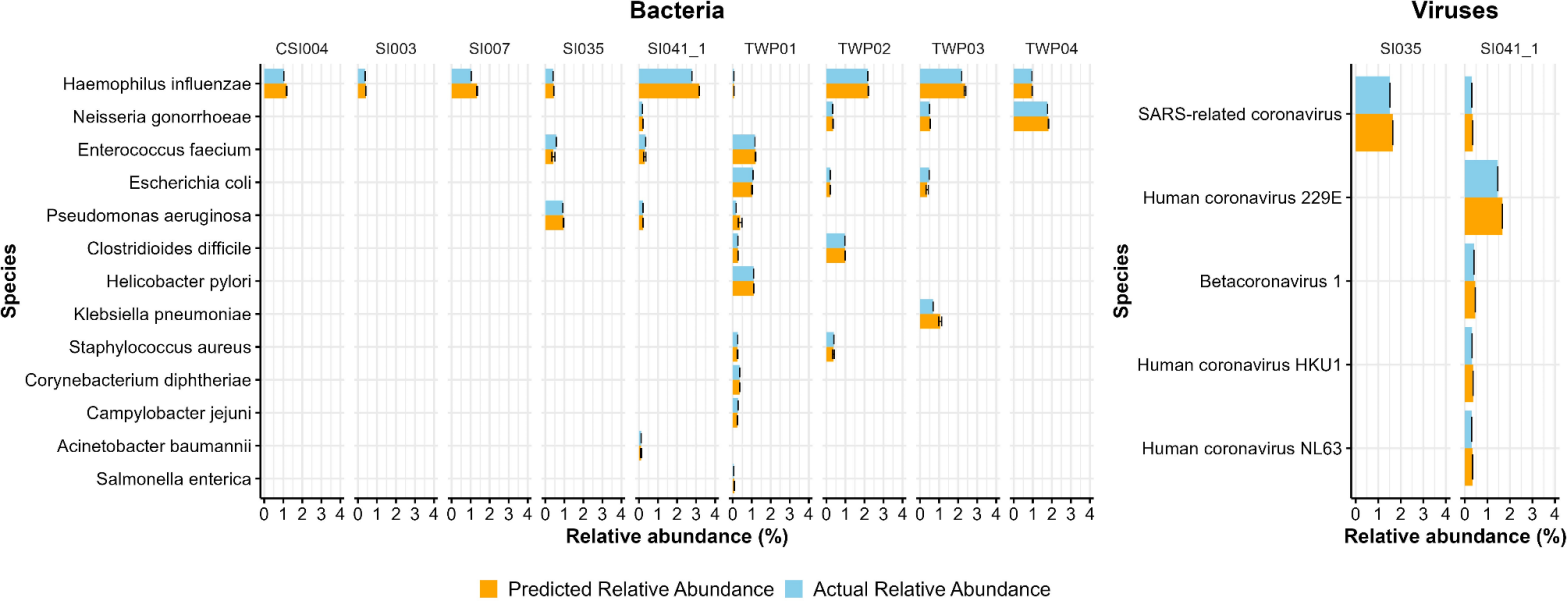
Comparison of actual and predicted relative abundances of critical bacterial and viral pathogens across multiple samples. The left panel shows bacterial taxa, while the right panel displays viral taxa. The distinct sample compositions are displayed side by side. In each one, the bars represent the mean relative abundance of a pathogen, and error bars indicate the standard deviation across the ten replicated samples of each composition

The pipeline’s ability to detect a wide range of respiratory pathogen markers was validated by benchmarking mock metagenomes with varying compositions. It identified over 177 out of 204 respiratory pathogens present in the composition. The pipeline demonstrated high accuracy in classifying critical pathogens. The predicted and actual relative abundances for bacterial pathogens were 7.3% different, with a standard deviation of 21.8%. For viral taxa, the mean difference was 13.2%, with a standard deviation of 2.4% (Fig. 2). Of the eighteen critical pathogens present in our samples, eleven had more than 85% concordance between their predicted and actual relative abundances.

### General taxa detection

In addition, for all taxa present in the samples, the pipeline successfully recovered 89% of them (420 out of 470), with a mean difference of only 5.8% and 17.5% standard deviation, between their predicted and actual relative abundances (Figures S1 and S2). Overall, the correlation between the expected and actual relative abundance for all taxa was approximately 0.9, with a statistically significant p-value of < 0.001 (Figure S3).

Out of the 420 recovered taxa, only 27 were underestimated or overestimated by more than 1.96 times the standard deviation (Figure S4). From these, three are classified as critical pathogens: *Pseudomonas aeruginosa*, *Klebsiella pneumoniae*, and *Salmonella enterica* (marked in red in Figure S4). These pathogens’ identification challenges are mainly due to their high genomic variability and similarity to other taxa, complicating their identification which is crucial for effective treatment and infection control.

### Empirical validation

In our analysis of the clinical dataset, we successfully identified all pathogens reported in the clinical diagnoses as the cause of infection. Despite the pipeline’s simplicity, it accurately detected most of the more abundant taxa, correctly identifying 46 out of 56 reported viral species and 273 out of 276 bacterial genera across the samples (Figures S5 and S6).

## Discussion

The proposed bioinformatics pipeline demonstrated robustness, reproducibility, and high accuracy in identifying and quantifying microbial taxa. It effectively identified a wide range of respiratory pathogens, making it a valuable tool for large-scale surveillance projects. The pipeline’s ability to accurately detect both abundant and rare taxa is crucial for monitoring pathogen dynamics and supporting early detection and risk modeling of infectious disease outbreaks. This aligns directly with country-wide surveillance initiatives, emphasizing the importance of robust surveillance systems in anticipating and managing public health threats [3].

A key advantage of our pipeline is its customizable and stable nature, which eliminates dependence on external updates and online services. This self-contained design ensures reliability and continuity, critical for large-scale projects where consistency is essential. Additionally, it is easily adaptable, allowing for the inclusion of additional genomes in the index databases to further enhance some relevant pathogen identification.

Overall, the pipeline’s methodological framework offers a consistent, customizable, and accurate tool for pathogen surveillance, enhancing the stability and effectiveness of large-scale public health systems. These attributes are critical for ensuring timely and effective responses to public health threats [13, 14].

### Limitations

A key limitation of our pipeline is the potential for false positives in taxonomic identification, particularly with closely related strains. This challenge, common in reference-based methods, arises from difficulties in distinguishing near-identical strains due to limited database diversity. The presence of such strains in samples can lead to misclassification and inaccurate estimation. Although our tests addressed this, the likelihood of encountering many in real-world surveillance is low. Expanding the reference database, especially with genomes of critical and hard-to-identify pathogens, could improve accuracy and reduce false positives. Our clinical analysis also revealed a few false positives, mainly in low-abundance taxa, likely due to the pipeline’s reliance on read-level classification, unlike the more complex genome assembly techniques used in the original study. This was particularly noticeable in viral identifications, highlighting the need to incorporate alternative approaches to further improve accuracy.

### Future directions

Future enhancements to the pipeline will aim to further improve its accuracy, scalability, and automation. Ongoing studies will refine its capabilities and explore new applications in pathogen surveillance. Potential improvements include enhancing the pipeline’s scalability and exploring new tools and approaches to further refine pathogen detection and AMR marker identification.

## Electronic supplementary material

Below is the link to the electronic supplementary material.

Supplementary Material 1

Supplementary Material 2

Supplementary Material 3

Supplementary Material 4

Supplementary Material 5

Supplementary Material 6

Supplementary Material 7

Supplementary Material 8

Supplementary Material 9

## Data Availability

Data and codes used in this study are available on the AESOP GitHub page (https://github.com/cidacslab/aesop-metagenomics-pipeline). The GRCh38 and T2T-CHM13v2.0 human reference genomes, were obtained from EMBL-EBI (http://ftp.ensembl.org/pub/release-112/fasta/homo_sapiens/dna/) and NCBI (http://ftp.ncbi.nlm.nih.gov/genomes/refseq/vertebrate_mammalian/Homo_sapiens/latest_assembly_versions/GCF_009914755.1_T2T-CHM13v2.0/), respectively. The Kraken2 Index is available at https://benlangmead.github.io/aws-indexes/k2 (we used the version updated on Jun 5th, 2024). The CZID pathogen list is available at https://czid.org/pathogen_list; the Illumina RPIP pathogen list in https://www.illumina.com/products/by-type/sequencing-kits/library-prep-kits/respiratory-pathogen-id-panel.html; the Illumina VSOP list in https://www.illumina.com/products/by-type/sequencing-kits/library-prep-kits/respiratory-pathogen-id-panel.html; the CDC outbreak reports in https://www.cdc.gov/outbreaks/index.html; and the WHO priority pathogen list reports in https://www.who.int/publications/i/item/9789240093461 and https://www.who.int/publications/m/item/pathogens-prioritization-a-scientific-framework-for-epidemic-and-pandemic-research-preparedness.

## Abbreviations

ÆSOP: Alert-Early System of Outbreaks with Pandemic Potential
HISAT2: Hierarchical Indexing for Spliced Alignment of Transcripts
DNA: Deoxyribonucleic Acid
LCA: Lowest Common Ancestor
Bracken: Bayesian Reestimation of Abundance with Kraken
FTP: File Transfer Protocol
EMBL-EBI: European Molecular Biology Laboratory - European Bioinformatics Institute
NCBI: National Center for Biotechnology Information
PFP: Protozoa, Fungi, and Plants
RefSeq: Reference Sequence database
bp: base pairs
QC: Quality Control
SAM: Sequence Alignment Map
MeSS: Metagenomic Sequence Simulator
CZID: Chan Zuckerberg Initiative Database
RPIP: Respiratory Pathogen ID/AMR Enrichment Panel
VSOP: Viral Surveillance and Outbreak Preparedness
CDC: Centers for Disease Control and Prevention
WHO: World Health Organization
SRA: Sequence Read Archive
HPC: High-Performance Computing
GB: Gigabyte
vCPU: virtual Central Processing Unit
RAM: Random Access Memory
AMR: Antimicrobial Resistance

## References

[1] Stephens ZD, Lee SY, Faghri F, Campbell RH, Zhai C, Efron MJ, et al. Big Data: Astronomical or Genomical? PLoS Biol. 2015;13:e1002195. 26151137 10.1371/journal.pbio.1002195 PMC4494865

[2] Goecks J, Nekrutenko A, Taylor J, Afgan E, Ananda G, Baker D et al. Galaxy: a comprehensive approach for supporting accessible, reproducible, and transparent computational research in the life sciences. Genome Biol. 2010;11. 10.1186/gb-2010-11-8-r86 PMC2945788 20738864

[3] Ramos PIP, Marcilio I, Bento AI, Penna GO, de Oliveira JF, Khouri R et al. Combining Digital and Molecular Approaches Using Health and Alternate Data Sources in a Next-Generation Surveillance System for Anticipating Outbreaks of Pandemic Potential. JMIR Public Health Surveill. 2024;10:e47673 https://publichealth.jmir.org/2024/1/e47673. 2024;10:e47673. 10.2196/47673 PMC10806444 38194263

[4] Chen S, Zhou Y, Chen Y, Gu J. Fastp: an ultra-fast all-in-one FASTQ preprocessor. Bioinformatics. 2018;34:i884–90. 30423086 10.1093/bioinformatics/bty560 PMC6129281

[5] Kim D, Paggi JM, Park C, Bennett C, Salzberg SL. Graph-based genome alignment and genotyping with HISAT2 and HISAT-genotype. Nat Biotechnol 2019. 2019;37:8. 10.1038/s41587-019-0201-4 PMC7605509 31375807

[6] Langmead B, Salzberg SL. Fast gapped-read alignment with Bowtie 2. Nature Methods 2012 9:4. 2012;9:357–9. 10.1038/nmeth.1923 PMC3322381 22388286

[7] Wood DE, Lu J, Langmead B. Improved metagenomic analysis with Kraken 2. Genome Biol. 2019;20:1–13. 31779668 10.1186/s13059-019-1891-0 PMC6883579

[8] Lu J, Breitwieser FP, Thielen P, Salzberg SL. Bracken: estimating species abundance in metagenomics data. PeerJ Comput Sci. 2017;2017:e104. 10.7717/peerj-cs.104 PMC12016282 40271438

[9] Li H, Handsaker B, Wysoker A, Fennell T, Ruan J, Homer N, et al. The sequence Alignment/Map format and SAMtools. Bioinformatics. 2009;25:2078. 19505943 10.1093/bioinformatics/btp352 PMC2723002

[10] Shen W, Ren H. TaxonKit: a practical and efficient NCBI taxonomy toolkit. J Genet Genomics. 2021;48:844–50. 34001434 10.1016/j.jgg.2021.03.006

[11] Gourlé H, Karlsson-Lindsjö O, Hayer J, Bongcam-Rudloff E. Simulating Illumina metagenomic data with InSilicoSeq. Bioinformatics. 2019;35:521–2. 30016412 10.1093/bioinformatics/bty630 PMC6361232

[12] Li CX, Li W, Zhou J, Zhang B, Feng Y, Xu CP et al. High resolution metagenomic characterization of complex infectomes in paediatric acute respiratory infection. Scientific Reports 2020 10:1. 2020;10:1–11. 10.1038/s41598-020-60992-6 PMC7054269 32127629

[13] Lindgreen S, Adair KL, Gardner PP. An evaluation of the accuracy and speed of metagenome analysis tools. Scientific Reports 2016 6:1. 2016;6:1–14. 10.1038/srep19233 PMC4726098 26778510

[14] Breitwieser FP, Lu J, Salzberg SL. A review of methods and databases for metagenomic classification and assembly. Brief Bioinform. 2019;20:1125–36. 29028872 10.1093/bib/bbx120 PMC6781581

