# Supplementary figures and images for: Design and implementation of a metagenomic analytical pipeline for respiratory pathogen detection
Source: BMC Res Notes. 2024 Oct 3;17:291. doi: 10.1186/s13104-024-06964-9 (PMC11451226; doi:10.1186/s13104-024-06964-9)

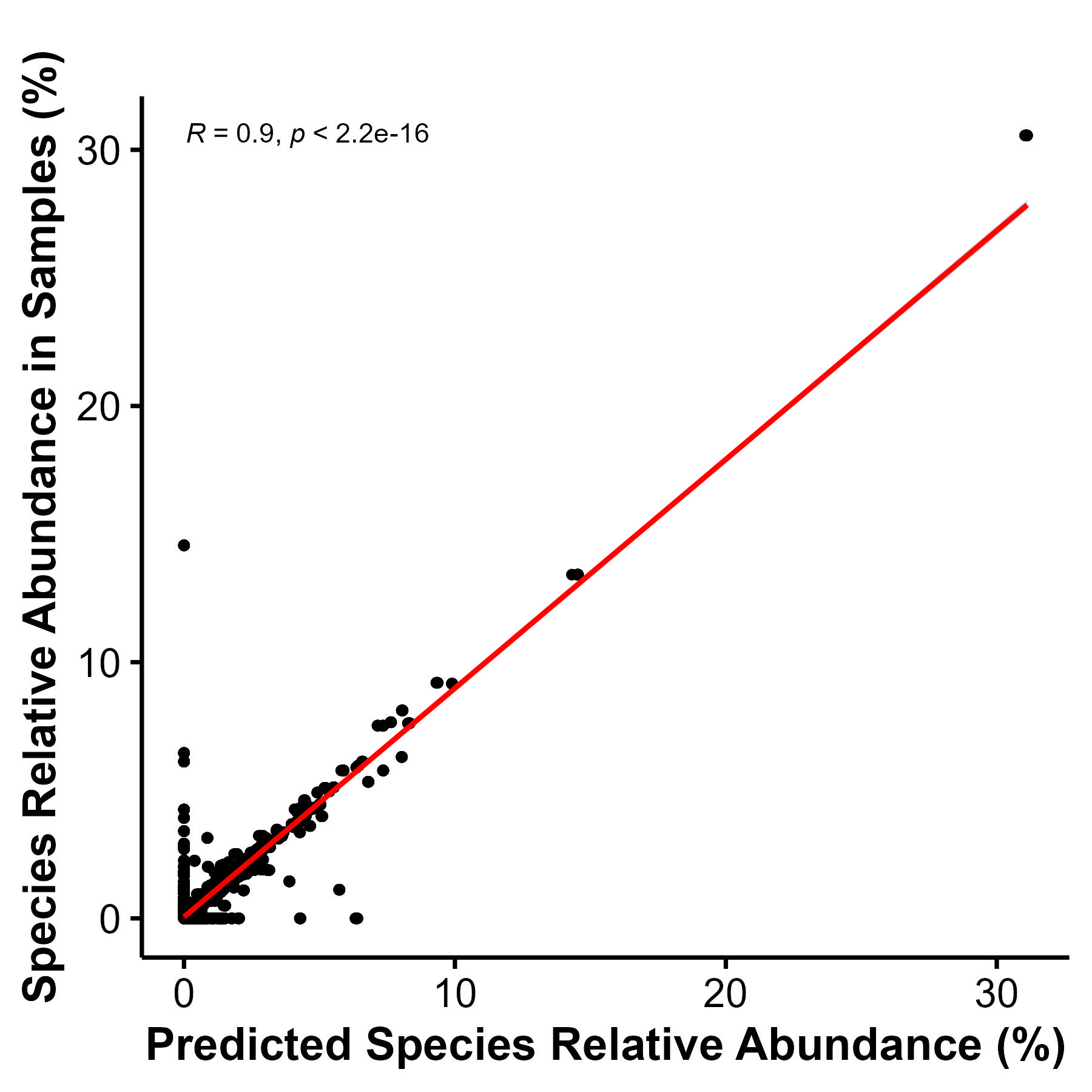

Supplement: Supplementary file 1 — Supplementary Material 1 [file 13104_2024_6964_MOESM1_ESM.jpg]

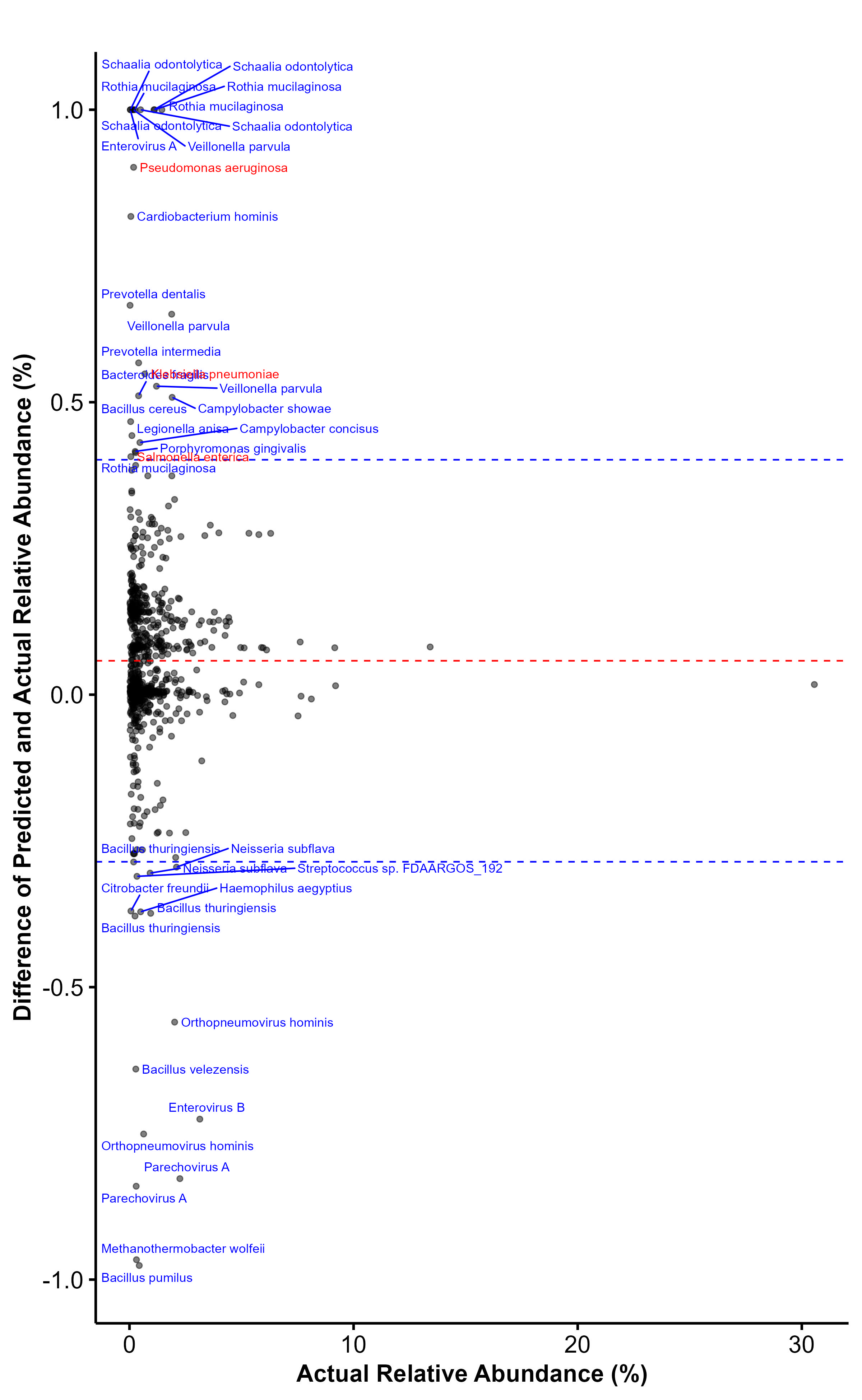

Supplement: Supplementary file 2 — Supplementary Material 2 [file 13104_2024_6964_MOESM2_ESM.jpg]

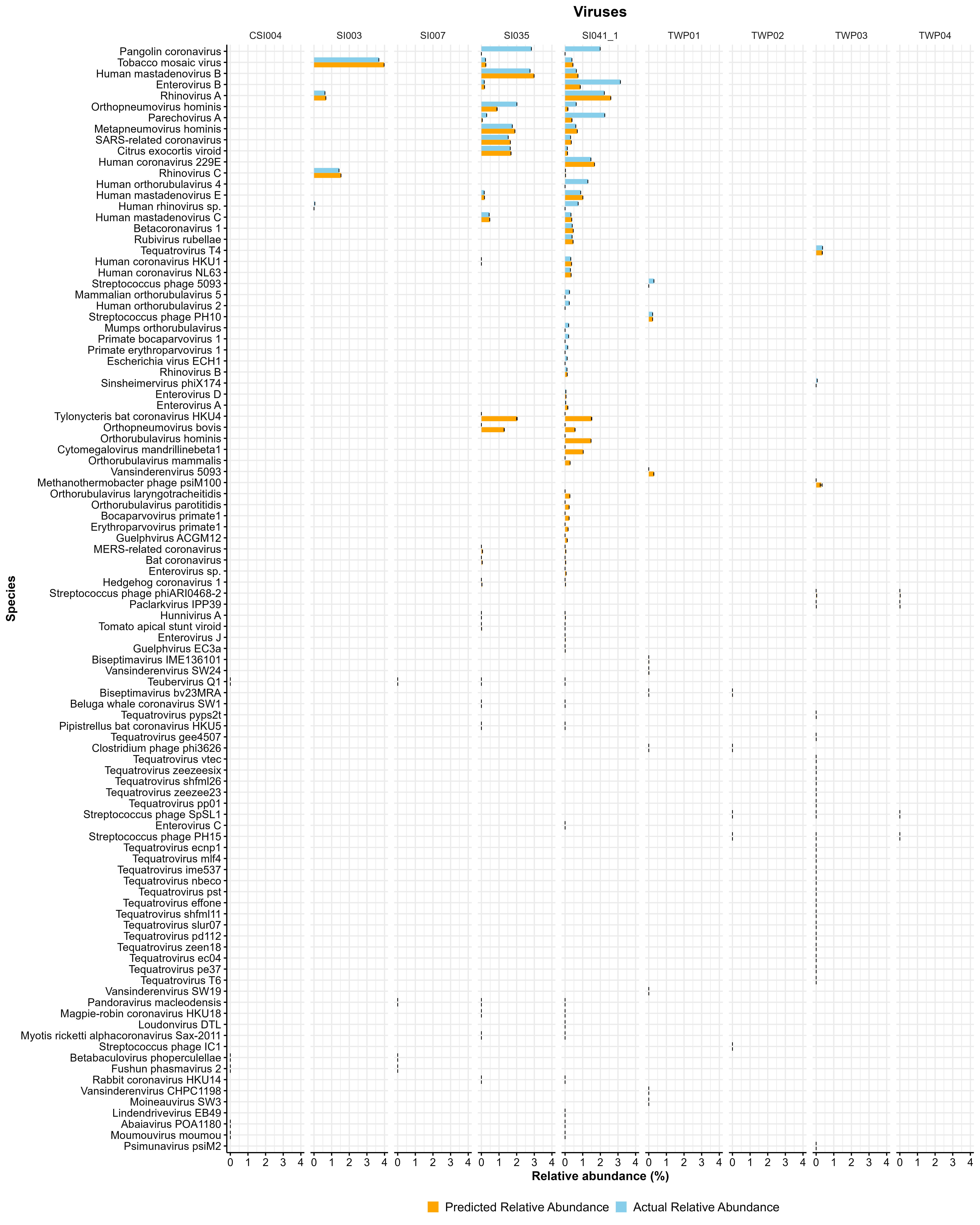

Supplement: Supplementary file 3 — Supplementary Material 3 [file 13104_2024_6964_MOESM3_ESM.jpg]

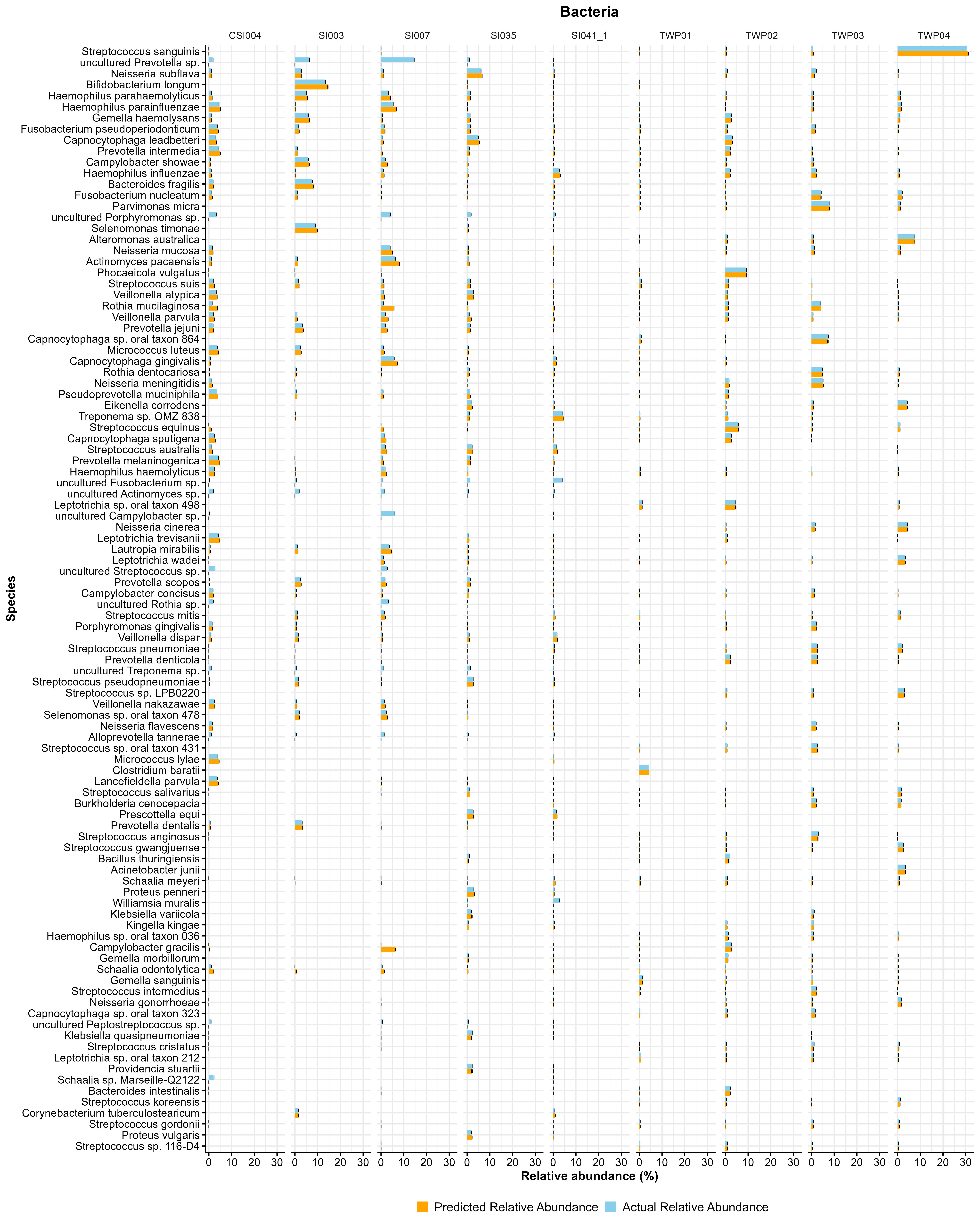

Supplement: Supplementary file 4 — Supplementary Material 4 [file 13104_2024_6964_MOESM4_ESM.jpg]

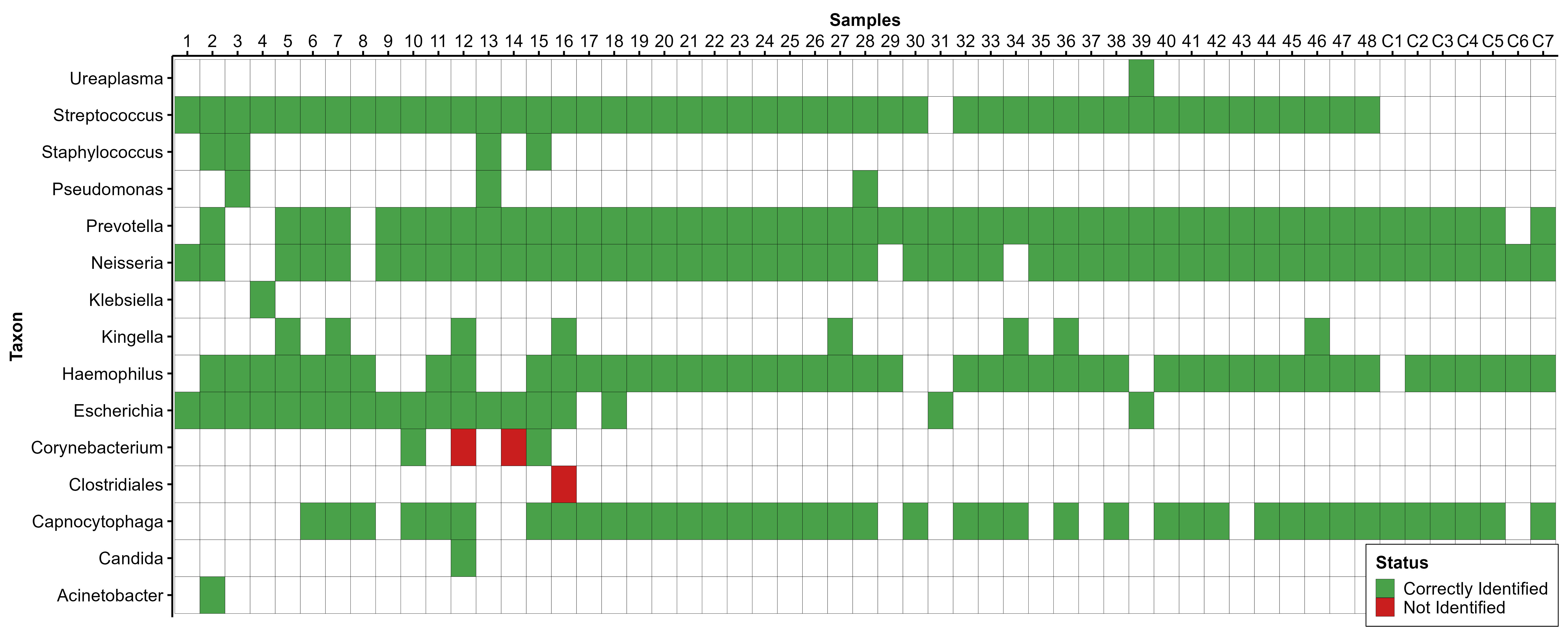

Supplement: Supplementary file 7 — Supplementary Material 7 [file 13104_2024_6964_MOESM7_ESM.jpg]

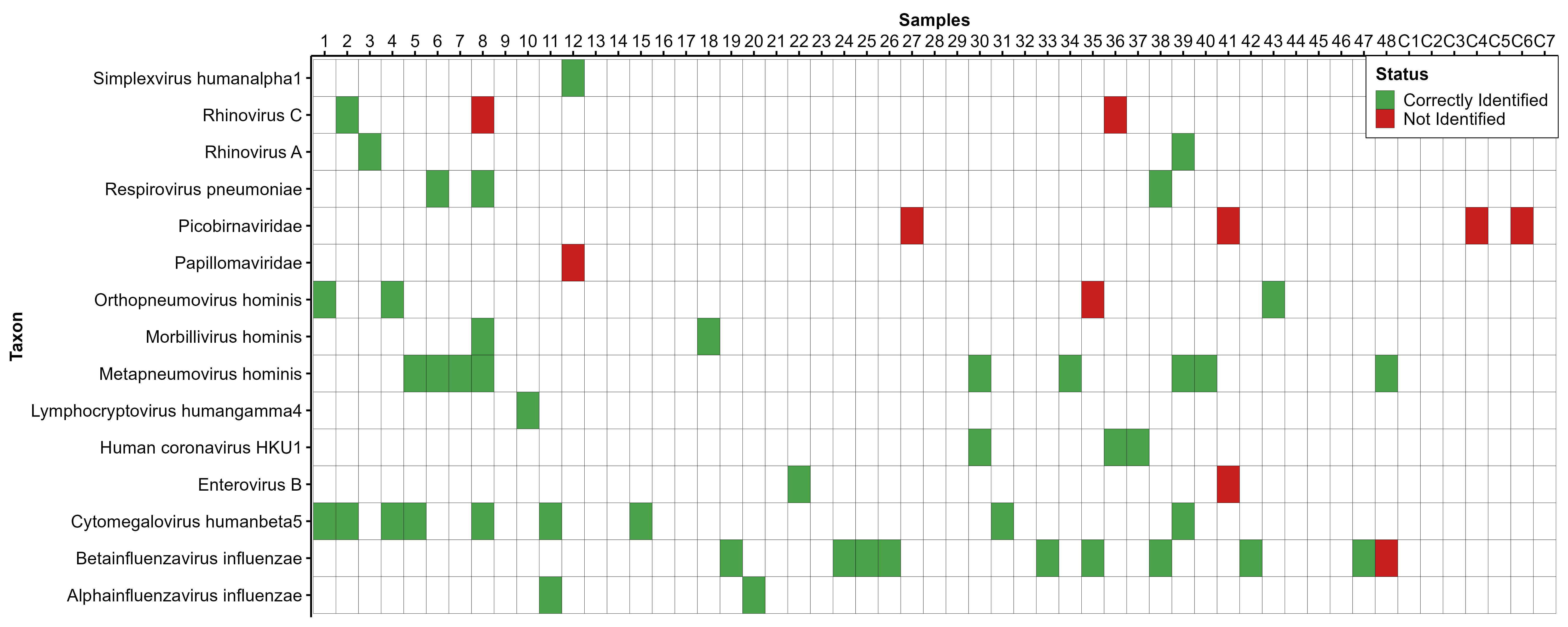

Supplement: Supplementary file 8 — Supplementary Material 8 [file 13104_2024_6964_MOESM8_ESM.jpg]
