# Supplementary material for: Design and implementation of a metagenomic analytical pipeline for respiratory pathogen detection
Source: BMC Res Notes. 2024 Oct 3;17:291. doi: 10.1186/s13104-024-06964-9 (PMC11451226; doi:10.1186/s13104-024-06964-9)
Supplement: Supplementary file 9 — Supplementary Material 9 [file 13104_2024_6964_MOESM9_ESM.docx]

# **Supplementary Information**

**Table S1** – Mock metagenomes composition.

*The tables related to mock composition are on GitHub at directory ‘data/pipeline_mock/composition’, containing the accession id from the NCBI of the genome and its corresponding abundance in the sample. The tables in the folder ‘data/pipeline_mock/metadata’ complement this information with data from the entire taxonomic hierarchy of each accession.*

**Table S2** - Metagenomic features.

Table is in file samples_genomic_features.xlsx

**Table S3** – Pathogen list and prioritization

Table is in file pathogen_prioritization.xlsx


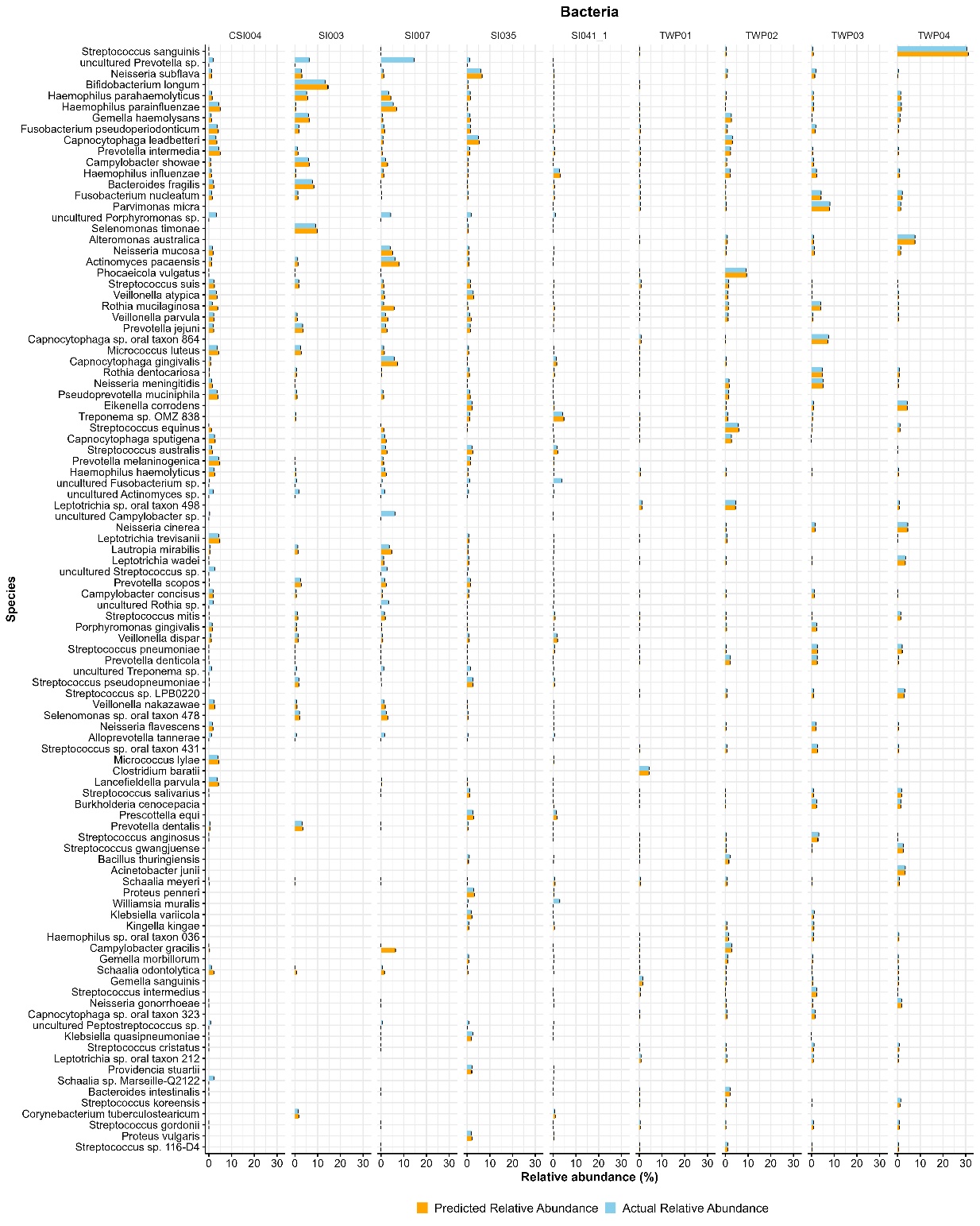


**Figure S1.** **Comparison of actual and predicted relative abundances of top 100 bacterial taxa across multiple samples**. The panel shows the 100 most abundant bacterial taxa in the samples. The distinct sample compositions are displayed side by side. In each one, the bars represent the mean relative abundance of the taxa and error bars indicate the standard deviation, across the ten replicated samples of each composition.


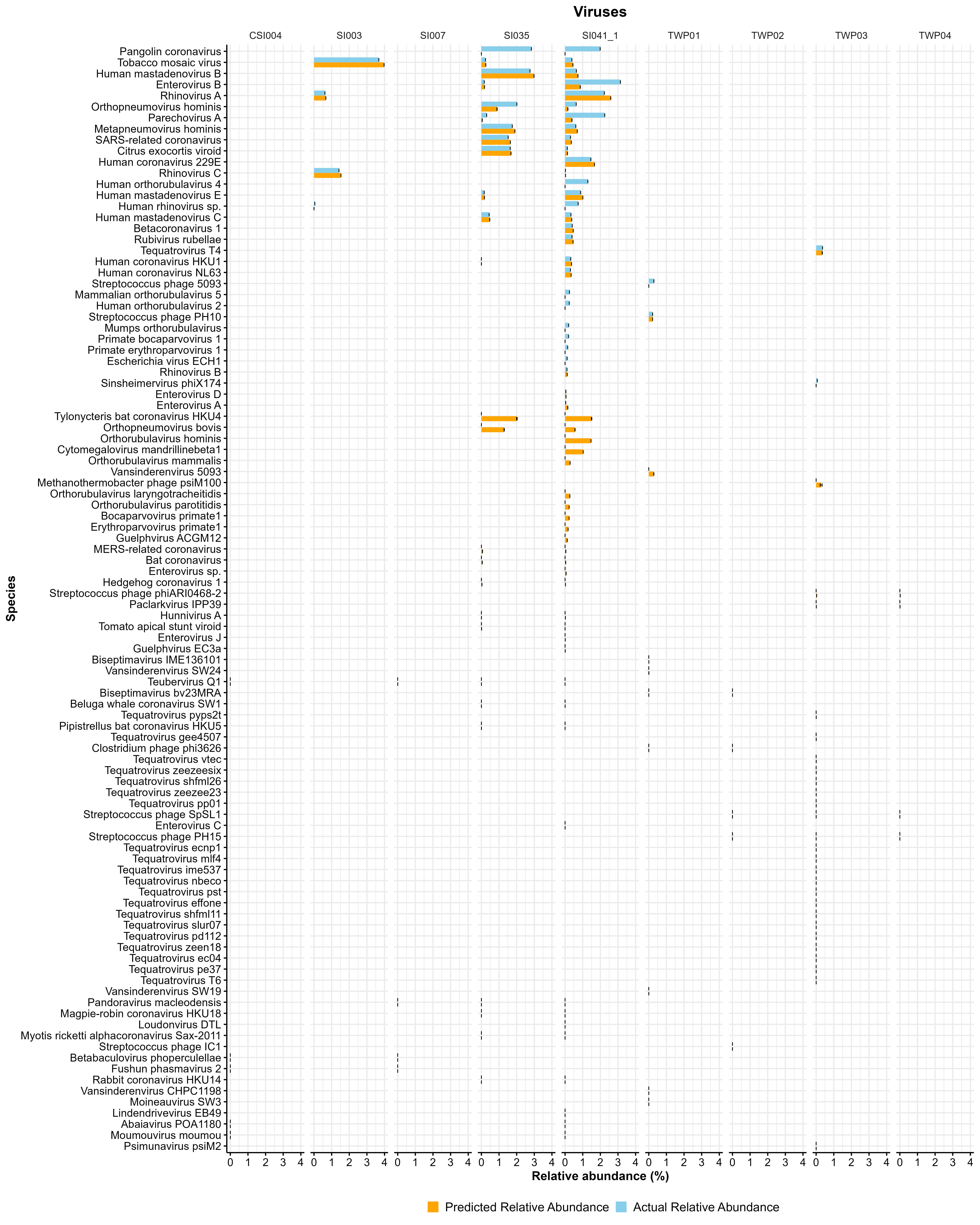


**Figure S2.** **Comparison of actual and predicted relative abundances of top 100 viral taxa across multiple samples**. The panel shows the 100 most abundant viral taxa in the samples. The distinct sample compositions are displayed side by side. In each one, the bars represent the mean relative abundance of the taxa and error bars indicate the standard deviation, across the ten replicated samples of each composition.


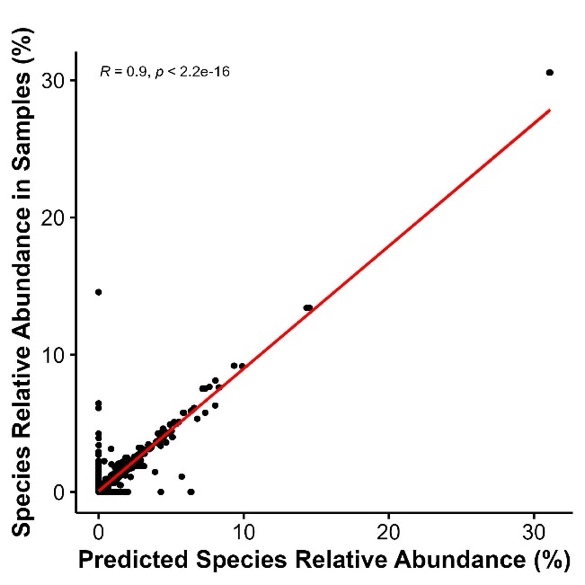


**Figure S3. Correlation between predicted and actual species relative abundance in metagenomic samples**. Each point represents a species in the samples, with the x-axis showing the predicted relative abundance and the y-axis showing the actual relative abundance. The red line represents the line of perfect agreement (y = x). The strong correlation (R = 0.9, p < 0.001) indicates a high level of accuracy in the pipeline's ability to predict species abundance.


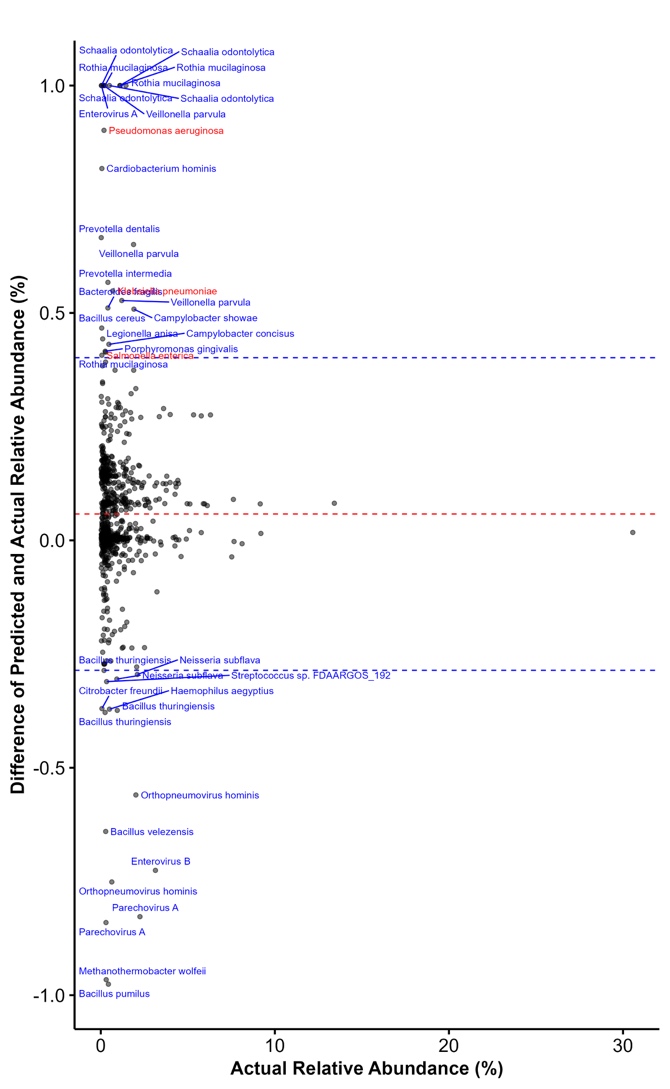


**Figure S4. Bland-Altman plot comparing the predicted and actual relative abundances of species in metagenomic samples.** The x-axis represents the actual relative abundance of species, while the y-axis shows the difference between predicted and actual relative abundance percentage relative to the actual abundance. The red dashed line indicates the mean difference (bias), and the blue dashed lines represent the limits of agreement (±1.96 standard deviations from the mean difference). Each point corresponds to a species in a sample. Species names are annotated for points that deviate significantly from the mean, highlighting potential outliers where the prediction was less accurate. Names of the key pathogens are written in red. This analysis helps identify species that were either overestimated or underestimated by the pipeline, providing insight into areas where predictive accuracy could be improved.


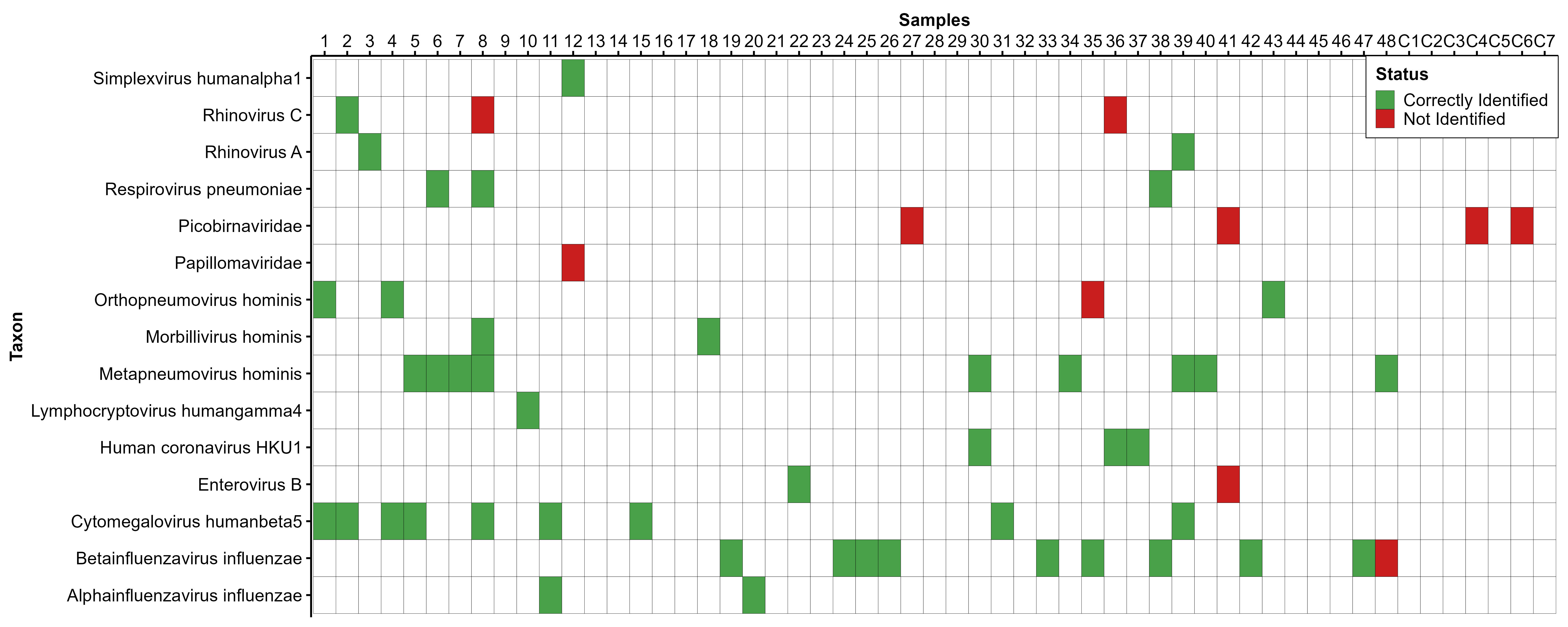


**Figure S5. Comparison of viral pathogen identification using the proposed pipeline on clinical dataset samples.** The heatmap shows the presence of identified taxa across 55 samples from the clinical dataset "High resolution metagenomic characterization of complex infectomes in paediatric acute respiratory infection" (Li et al., 2020). We highlighted the viral taxa identified in the original study. Green tiles indicate taxa we correctly identified by our pipeline. While red tiles highlight taxa that were not identified by our pipeline. The analysis shows that most pathogens were successfully identified, with some gaps in identification corresponding to low-abundance taxa. Despite that, all pathogens that were described as the cause of the infection were correctly identified by our pipeline.


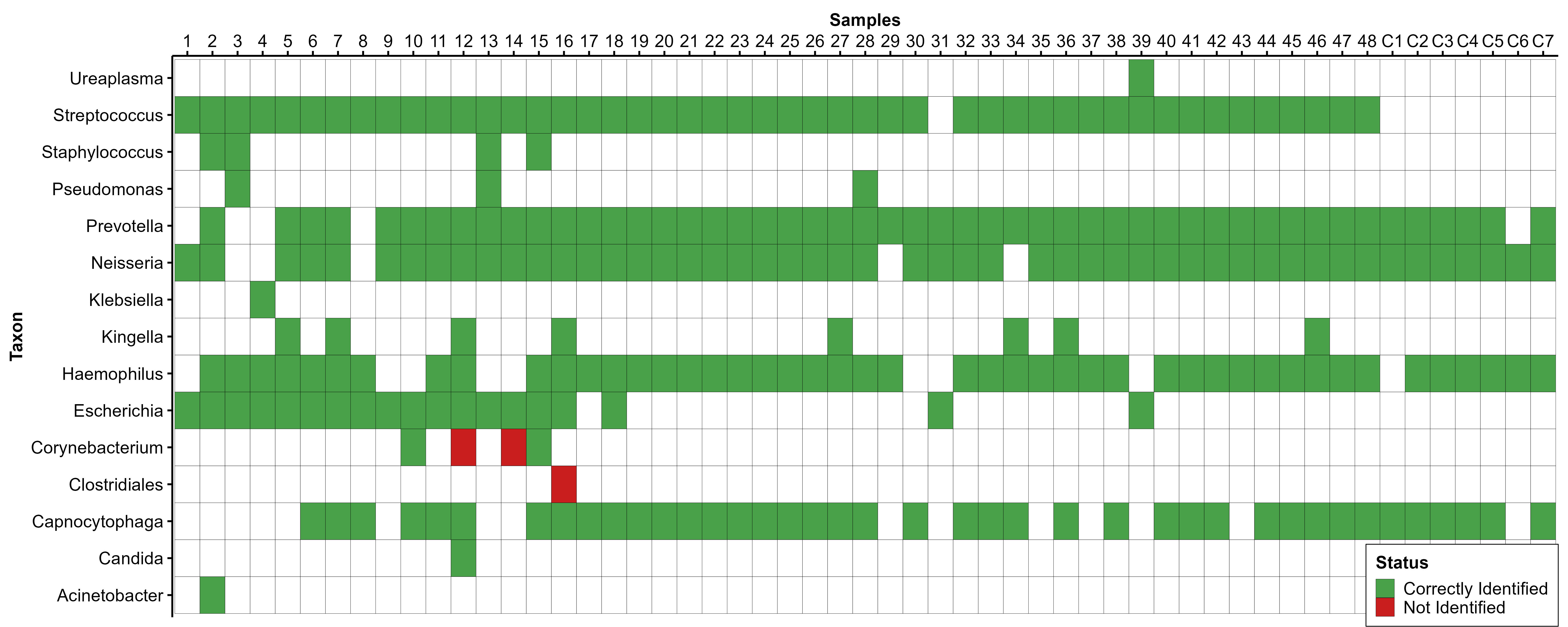


**Figure S6. Comparison of bacterial genera pathogen identification using the proposed pipeline on clinical dataset samples.** The heatmap shows the presence of identified taxa across 55 samples from the clinical dataset "High resolution metagenomic characterization of complex infectomes in paediatric acute respiratory infection" (Li et al., 2020). We highlighted the bacterial taxa identified in the original study. Green tiles indicate taxa we correctly identified by our pipeline. While red tiles highlight taxa that were not identified by our pipeline. The analysis shows that most pathogens were successfully identified, with some gaps in identification corresponding to low-abundance taxa. Despite that, all pathogens that were described as the cause of the infection were correctly identified by our pipeline.
